# The relationship between impostor phenomenon and career decision-making difficulties among nursing interns: the mediating role of psychological resilience

**DOI:** 10.3389/fpsyg.2024.1484708

**Published:** 2024-11-27

**Authors:** Meina He, Yao Li, Hui Hu, Zuqiang Yu, Cong Cai, Yao Cheng, Lu Ma, Shanshan Liu

**Affiliations:** ^1^School of Medical Humanities, Hubei University of Chinese Medicine, Wuhan, China; ^2^Hubei Shizhen Laboratory, Wuhan, China; ^3^School of Nursing, Hubei University of Chinese Medicine, Wuhan, China; ^4^School of Transportation and Logistics Engineering, Wuhan University of Technology, Wuhan, China; ^5^Department of Internal Neurology of Wuhan Fourth Hospital, Wuhan, China; ^6^Department of Nursing, Hubei Provincial Hospital of Traditional Chinese Medicine, Wuhan, China; ^7^Department of Gastroenterology, Tongji Hospital Affiliated to Tongji Medical College of Huazhong University of Science and Technology, Wuhan, China; ^8^Department of Gastroenterology, Zhongnan Hospital of Wuhan University, Wuhan, China

**Keywords:** nursing interns, impostor phenomenon, career decision-making difficulties, psychological resilience, mediating role

## Abstract

**Background:**

In the face of a global nursing shortage, nursing interns are crucial to sustaining the healthcare workforce. However, these interns encounter significant challenges in career decision-making, often exacerbated by the impostor phenomenon, which impedes their capacity to make informed choices. Despite its importance, little research has been reported on the specific conditions and contributing factors that affect nursing interns’ career decision-making processes.

**Objective:**

To understand the current status of nursing interns’ impostor phenomenon, psychological resilience, and career decision-making difficulties, and to validate the mediating role of psychological resilience between impostor phenomenon and career decision-making difficulties using structural equation modeling.

**Methods:**

Five hundred eighty-two nursing interns from four tertiary hospitals in Wuhan City, Hubei Province of China were selected by stratified random sampling. Data were collected with the Clance Impostor Phenomenon Scale, Career Decision-Making Difficulties Questionnaire, and the 10-item Connor–Davidson Resilience Scale through an online survey. Mediation effect tests were conducted using SPSS 26.0 and PROCESS software.

**Results:**

(1) The score for career decision-making difficulties was 99.34 (SD = 21.78), the score for impostor phenomenon was 57.58 (SD = 12.13), and the score of psychological resilience was 32.11 (SD = 8.50); (2) Psychological resilience had a significant negative correlation with career decision-making difficulties (*r* = −0.724, *p* < 0.01), and impostor phenomenon had a significant negative correlation with psychological resilience (*r* = −0.608, *p* < 0.01), and had a significant positive correlation with career decision-making difficulties (*r* = 0.700, *p* < 0.01). Psychological resilience played a partial mediating role between impostor phenomenon and career decision-making difficulties (*p* < 0.01), with the mediating effect (Effect Value = 0.518, Bootstrap 95% CI: 0.430, 0.610) accounting for 41.27% of the total effect.

**Conclusion:**

Career decision-making difficulties are common among nursing interns. Effective strategies should address the impostor phenomenon and promote psychological resilience to enhance self-awareness and create a supportive environment.

## 1 Introduction

According to a report from the World Health Organization (WHO), the global shortage of healthcare workers will reach 12.9 million by 2035 (Liaw et al., 2017). The regions anticipated to experience the most severe shortages are Africa and the Eastern Mediterranean, with projected reductions of 7 and 15%, respectively (Boniol et al., 2022). The nursing profession, in particular, is witnessing a higher attrition rate than the influx of new entrants (Adams et al., 2021), leading to a persistent shortage of nurses even in developed nations such as the United States and Canada (Baker, 2022; Tomblin Murphy et al., 2022). By the end of 2022, the total number of registered nurses in China had surpassed 5.2 million. However, this figure still needs to be improved to meet the demand for the nursing profession (Su et al., 2023). Nurses are pivotal in the healthcare system and constitute the largest segment of healthcare professionals. The current shortage of nursing personnel poses significant challenges to the global healthcare system, potentially affecting the allocation of healthcare resources, the quality of healthcare services, and the capacity to effectively respond to public health emergencies (Tamata and Mohammadnezhad, 2023). The government and relevant institutions must implement strategies to increase the attractiveness and entry rates into the nursing profession to address this issue effectively. Although research has been conducted on promoting nurses’ career success and professional identity to reduce turnover rates (Xue et al., 2022; Meng et al., 2023), there is a need to shift the focus of early career promotion interventions toward nursing interns who are making career choices. In the context of nursing education at Chinese universities, all nursing students must complete a minimum of 8 months of clinical practice in public hospitals before graduating as fully qualified nurses. However, internship experiences may undermine the career commitment of nursing students (Zhao et al., 2022). Nursing students may hesitate or choose not to enter the nursing profession if they find it difficult to make a career choice. This difficulty may result from insufficient identification with the nursing profession, low career selection self-efficacy, or low professional identity. These factors may lead potential nurses to choose alternative career paths, thus exacerbating the problem of nurse shortages. In the realm of healthcare, nursing interns stand at the crossroads of their professional journey, confronted with pivotal career decisions that will shape their future trajectories. Therefore, it is essential to emphasize the importance of making informed career decisions that will facilitate their transition into full-time nursing roles by the conclusion of their clinical practice.

Career decision-making difficulties encompass the challenges individuals encounter when confronted with career choices (Gati et al., 2010). According to the classification system established by Gati et al. (1996), five distinct types of career decision-making difficulties have been identified: unmotivated, generally indecisive, unrealistic, uninformed, and conflicted (Levin et al., 2022). Nursing students often face several challenges when making career decisions, including the choice of specialization (such as clinical nursing, nursing management, or academic research), selection of internship placements, employment locations (urban or rural), and work environments (hospitals, clinics, or community care). Multiple studies have shown that students prefer to work in hospitals rather than community hospitals. In terms of department preferences, they are inclined to work in pediatrics, emergency departments, and operating rooms, while psychiatry and geriatrics are the least popular. Their choices are often influenced by factors such as the work environment, salary levels, career development opportunities, and the flexibility of working hours (Cheng et al., 2015; Matarese et al., 2019; Sela et al., 2020). The process of career decision-making is complex and involves various factors such as self-concept, learning experiences, comparing alternatives, and implementing choices. Moreover, personal values, family background, and social environment significantly influence this process (Kulcsár et al., 2020). In Asian cultures, students’ career decision-making processes may be influenced by the expectations of parents and other authority figures (Sari and Putra, 2019). College students face multiple challenges in career decision-making, including self-assessment, addressing weaknesses, enhancing confidence, and developing potential (Abdullah, 2019). Various factors, including workload, nurse–patient relationships, career advancement opportunities, burnout, and risks of infection, consistently influence the professional environment for nurses (Admi et al., 2018). These elements exacerbate nursing interns’ distress during their career decision-making process (Chen et al., 2019; Anyango et al., 2024a). Nursing interns often find making informed career choices particularly daunting due to their limited understanding of the nursing field (Wang, 2023). The clinical practice serves as an essential phase for nursing students, facilitating the development of professional values, informed career choices, and a smooth transition into the nursing field (Tseng et al., 2013). This period allows educators to evaluate interns’ career decisions and apply appropriate interventions (Anyango et al., 2024b). However, few studies have focused on the career decision-making difficulties of nursing interns (Roush et al., 2021).

The impostor phenomenon was first defined by Clance and Imes (1978). They initially observed this phenomenon within a group of highly educated women. Despite their notable academic and professional achievements, these women frequently believe they lack genuine intelligence and have merely deceived others about their capabilities (Harvey and Katz, 1985). It describes a psychological experience where individuals doubt their accomplishments and fear being exposed as a “fraud.” These individuals believe that their abilities are overestimated by others and fear being exposed, despite having a consistent record of success. They tend to downplay praise directed toward them, are critical of their own achievements, and attribute their success to external factors rather than their own ability or intelligence (Mak et al., 2019). This issue is particularly pronounced among nursing interns, where women are more likely than men to experience the impostor phenomenon, indicating that this demographic is at a heightened risk for anxiety and diminished self-confidence (Peng et al., 2022). Individuals experiencing the impostor phenomenon find themselves caught in a challenging cycle difficult to break (Para et al., 2024). The positive emotions quickly fade, replaced by anxiety, procrastination, or excessive preparation. In turn, it fuels self-doubt even more (Jacobs and Sasser, 2021). Consequently, for those affected by this phenomenon, new achievements reinforce the belief of deceiving others rather than boosting self-confidence (Walker and Saklofske, 2023). Moreover, individuals experiencing the impostor phenomenon are at a higher risk of detrimental effects on health and well-being in the workplace. They often exhibit lower job satisfaction (Vergauwe et al., 2015) and face challenges in maintaining a healthy work-life balance (Crawford et al., 2016). This condition frequently contributes to mental health issues, including depression, anxiety, and low self-esteem (Kananifar et al., 2015; Neureiter and Traut-Mattausch, 2016; Leonhardt et al., 2017). The impostor phenomenon can induce anxiety, depression, and diminished confidence in career choices, potentially leading to suboptimal decisions or reluctance to pursue certain paths due to fear of failure or exposure. However, current research on career decision-making difficulties lacks exploration from the perspective of impostor phenomenon. Addressing the impostor phenomenon is a crucial step toward enhancing workplace health, employee fulfillment, and vocational development (Chen et al., 2022; Pignault et al., 2023; Gullifor et al., 2024).

Psychological resilience is an individual’s capacity to maintain a positive mental state during adversity, trauma, or significant stress, exemplifying substantial adaptability (Troy et al., 2023). The ability to maintain positive emotions and behaviors in difficult situations may be influenced by genetic factors that contribute to individual differences in responses and attitudes toward the environment (Smeeth et al., 2021). Personality traits, positive emotions, coping styles, and flexibility in emotional regulation are critical predictors of psychological resilience trajectories (Bonanno and Diminich, 2013). Psychological resilience also includes specific strengths and weaknesses in domains such as academics, career performance, and physical health (Denckla et al., 2020). Research has confirmed the effectiveness of psychological resilience in maintaining healthcare workers’ mental health (Labrague, 2021). The emotional attributes of self-assurance, hopefulness, optimism, and resilience exhibited by psychological resilience are crucial factors that support successful career decision-making (Zhou A. et al., 2024). This is because emotions significantly influence challenges associated with career decision-making (Farnia et al., 2018). Positive emotions generally reduce the likelihood of encountering these difficulties, while negative emotions like anxiety and low self-esteem can undermine career self-efficacy (Farnia et al., 2018; Yi et al., 2022). Psychological resilience is a critical attribute that significantly shapes an individual’s career identity (Yi et al., 2023). It allows individuals to navigate workplace adversities with greater efficacy, thereby fostering a deeper commitment to their professional roles. This resilience acts as a buffer against the onset of burnout symptoms, which can be particularly debilitating in high-stress environments (Guo et al., 2018). Regarding college students, the impact of psychological resilience is profound during the critical phase of career exploration (Lyu et al., 2022). Nursing interns, who are under significant stress and pressure during their internships, can benefit from resilience in navigating the challenges they face and in making career decisions. Psychological resilience is not merely a trait; it is a multifaceted construct that includes cognitive, emotional, and behavioral components. It is a process that can be developed and strengthened over time through various interventions and personal efforts (Chen et al., 2023). This is in contrast to the impostor phenomenon, which is often viewed as a more stable internal experience of self-doubt and fraudulence (Neureiter and Traut-Mattausch, 2017). In this context, psychological resilience emerges as a crucial factor that may mediate the relationship between the impostor phenomenon and career decision-making difficulties. Psychological resilience, defined as the ability to adapt and bounce back from adversity, can buffer the negative effects of the impostor phenomenon and facilitate more effective career decision-making.

Considering the importance of impostor phenomenon, and psychological resilience on the career decision-making difficulties of nursing interns, this study investigated the impostor phenomenon, psychological resilience, and the career decision-making difficulties levels of nursing interns in Chinese hospital, aiming to explore the relationship between the three variables, and the mediating effect of psychological resilience between the other two variables, put forward some suggestions for improving the psychological resilience, and promoting their career decision-making of nursing interns, to provide empirical support for educational managers to take effective, and comprehensive measures to improve the career decision-making difficulties from the perspective of psychological resilience, which is an internal psychological resource. By understanding this dynamic, we can develop targeted interventions to support nursing interns in overcoming the impostor phenomenon and making informed career decisions. This research is not only timely but also essential for nurturing a confident and competent nursing workforce that can meet the challenges of the future. Therefore, this study puts forward the following hypotheses:

*Hypothesis 1*: Nursing interns’ impostor phenomenon is associated with psychological resilience.

*Hypothesis 2*: Nursing interns’ psychological resilience is associated with career decision-making difficulties.

*Hypothesis 3*: Nursing interns’ impostor phenomenon is associated with career decision-making difficulties.

*Hypothesis 4*: Psychological resilience mediates the relationship between impostor phenomenon and career decision-making difficulties.

## 2 Methods

### 2.1 Study design and participants

This study was conducted as a cross-sectional analysis utilizing an online survey questionnaire between June 2024 and August 2024. Participants were recruited through convenience sampling at four tertiary hospitals in Wuhan, Hubei Province of China. This period was chosen as it corresponds with the mid-to-late stages of the participants’ internships, which is a critical phase for career decisions. Therefore, their experiences and insights are precious for this research. Informed consent was obtained before their involvement to ensure voluntary participation and the protection of participants’ rights. The inclusion criteria for this study were: (1) active nursing interns; (2) at least 6 months of internship experience; (3) able to understand the content of the questionnaire; (4) aged 18 or above; (5) cooperation with the investigators. The exclusion criteria for this study were: (1) absence of clinical internship experience; (2) inability to comprehend the questionnaire; (3) non-cooperation with the investigators.

The sample size was calculated according to the principle of Kendall’s estimation of sample size. It demonstrated that the sample size was 5–10 times that of the independent variables. This study included 21 independent variables. The general demographic information questionnaire included 5 social demographic variables, while the 3 questionnaire scales consisted of 16 dimensions, resulting in a total of 21 variables. The calculation formula was *N* = (5 + 3 + 3 + 10) * 10 = 210. Considering a 20% sample loss rate, the minimum sample size was 252 cases. A total of 605 participants participated in the study. Five hundred and eighty two valid questionnaires were finally collected, with a valid recovery rate of 96.19%, which satisfied the minimum sample size required for this study.

### 2.2 Questionnaire tool

#### 2.2.1 Participants’ socio-demographic characteristics

The social demographic characteristics of the participants included age, gender, registered residence, internship duration, and clear career intentions.

#### 2.2.2 Impostor phenomenon

Impostor phenomenon was measured using the Chinese version of the Clance Impostor Phenomenon Scale (CIPS), revised by Jiang et al. (2022). The scale consists of 18 items across three dimensions: fake (4 items), luck (6 items), and discount (8 items). “Fake” indicates self-doubt and concerns about ability; “luck” indicates the attribution of successes to luck; “discount” indicates the inability to internalize success and praise (Chrisman et al., 1995). The responses were provided on a 5-point Likert scale ranging from (1) “not at all true” to (5) “very true”. The total score range from 18 to 90, with higher score indicates a greater severity of impostor phenomenon. The scale’s Cronbach’s alpha was 0.930. In this study, Cronbach’s alpha was 0.881, and Cronbach’s alpha of each dimension was between 0.747 and 0.844.

#### 2.2.3 Career decision-making difficulties

Career decision-making difficulties were measured using the Chinese version of the Career Decision-making Difficulties Questionnaire (CDDQ), revised by Shen (2005). The questionnaire consists of 35 items across three dimensions: lack of readiness (10 items), lack of information (12 items), and inconsistent information (10 items). In addition, 3 screening items were not included in the total score. The responses were provided on a 5-point Likert scale ranging from (1) “strongly disagree” to (5) “strongly agree.” A higher scores indicating greater difficulties in career decision-making. The questionnaire’s Cronbach’s alpha was 0.866. In this study, Cronbach’s alpha was 0.919, and Cronbach’s alpha of each dimension was between 0.815 and 0.919.

#### 2.2.4 Psychological resilience

Psychological resilience was measured using the 10-item Connor–Davidson Resilience Scale (CD-RISC-10) by Campbell-Sills and Stein (2007). Chinese scholars (Cheng et al., 2020) have conducted a validation study on this scale among undergraduates, which demonstrated good reliability and validity including strong internal consistency and criterion-related validity. The scale consists of 10 items. The responses were provided on a 5-point Likert scale ranging from (0) “never” to (4) “always.” A higher score indicates a better psychological resilience. The scale’s Cronbach’s alpha was 0.85. In this study, the Cronbach’s alpha was 0.895.

### 2.3 Data collection

The questionnaires for nursing interns were administered through the online platform “Wenjuanxing.” Before distribution, we collaborated with the heads of the nursing departments at the participating hospitals to secure their cooperation. These leaders facilitated the mobilization and guidance of interns across various departments to ensure the completion of the questionnaires. Participants anonymously completed the questionnaires by scanning a WeChat QR code. The completion of all questions was required before submitting the questionnaire. Scanning the QR code and completing the questionnaire signified consent to participate in this study. Measures were implemented to ensure meticulous and accurate responses. For example, responses completed within less than 2 min or those displaying clear patterns were excluded from the analysis. The questionnaire was administered with informed consent while maintaining anonymity. A detailed explanation of the purpose and significance of this study is provided on its front page to ensure the impartiality of the data.

### 2.4 Data analysis

The Statistical Package for the Social Sciences (SPSS) software (v26) (IBM Corp., Armonk, NY, United States) was used in all analyses. As all survey items required responses, there was no need to address missing values. We calculated descriptive statistics for social demographic information, impostor phenomenon, career decision-making difficulties, and psychological resilience. Statistical descriptions of count data were presented as component ratios [*n*(%)], normally distributed measurement data as (mean ± SD). Independent sample t-tests or one-way ANOVA were used to compare statistical differences in career decision-making difficulties with different socio-demographic characteristics. Pearson’s r was calculated to test the relationships between nursing interns’ impostor phenomenon, career decision-making difficulties, and psychological resilience. Multiple linear regression analysis was used to explore the mediating effects of psychological resilience. Finally, the Bootstrap method (5,000 iterations) was applied by running the PROCESS plugin in the SPSS Macro to test the statistical significance of the mediating effect. If the 95% confidence interval did not contain 0, the mediating effect was significant. In all analyses, statistically significant was set at *p* < 0.05.

### 2.5 Ethical considerations

This study protocol was approved by the Medical Research Ethics Committee at Hubei University of Traditional Chinese Medicine (No. 2024010). Following the confidentiality norms and protocols outlined in the Declaration of Helsinki, potential participants were informed about the study’s objectives and procedures through a web-based platform, emphasizing their voluntary participation. Participants provided informed consent voluntarily. All collected data were kept confidential and anonymous to protect participant privacy. The study was designed and executed with transparency, objectivity, and integrity in data collection and analysis processes.

## 3 Results

### 3.1 Common method bias test

This study used self-reported data, which may lead to common method bias. Harman’s single-factor test (Podsakoff et al., 2012) was employed to assess this potential bias. The results revealed eight common factors, each with an eigenvalue more significant than one. Notably, the first factor accounted for only 32.9% of the variance, which is below the critical threshold of 40%, suggesting that common method bias is not a significant concern in this study.

### 3.2 General characteristics of the study

According to the Table 1, 479 of the participants are women (82.3%) and 103 (17.7%) are men. The mean age of the 582 participants was 21.32 (SD = 1.371) years, with a range from 19 to 24 years old. 275 (47.3%) of the participants come from the urban and 307 (52.7%) come from rural. 415 (71.3%) of the participants have clear career intentions and 167 (28.7%) lack clear career intentions during the internship.

**Table 1:** Participants characteristics and differences of career decision-making difficulties among groups (*n* = 582).

Variable	Group	Frequency (percentage)	Career decision-making difficulties (mean ± SD)	*t*/*F*	*p*
Gender	Men	103 (17.7)	95.43 ± 24.87	4.066	0.044
	Women	479 (82.3)	100.18 ± 20.99		
Age (years)	19	59 (10.1)	96.59 ± 25.09	0.995	0.420
	20	100 (17.2)	97.47 ± 21.72		
	21	171 (29.4)	98.20 ± 21.22		
	22	147 (25.3)	100.95 ± 21.06		
	23	55 (9.4)	101.62 ± 23.35		
	24	50 (8.6)	103.02 ± 19.76		
Registered residence	Urban	275 (47.3)	96.60 ± 21.89	8.392	0.004
	Rural	307 (52.7)	101.80 ± 21.42		
Internship duration	Less than 8 months	305 (52.4)	97.26 ± 22.15	0.682	0.563
	8–9 months	195 (33.5)	99.92 ± 21.63		
	More than 9 months	82 (14.1)	101.00 ± 21.91		
Clear career intentions	Yes	415 (71.3)	96.34 ± 22.03	28.802	0.000
	No	167 (28.7)	106.80 ± 19.27		

Among the 582 surveyed participants, women nursing interns from rural areas who lacked clear career intentions during the internship had significantly higher scores for career decision-making difficulties (all *p* < 0.05). Age and duration of internship were not significantly associated with career decision-making difficulties.

### 3.3 Current status level of the three variables

As listed in Table 2, the total score of career decision-making difficulties was 99.34 (SD = 21.78), and the mean score of each item was 3.10 (SD = 0.68). “Lack of information” had the highest score for the three dimensions of career decision-making difficulties. The total score of impostor phenomenon was 57.58 (SD = 12.13), and the mean score of each item was 3.20 (SD = 0.67). “Fake” had the highest score for the three dimensions of impostor phenomenon. The total score of the psychological resilience was 32.11 (SD = 8.50), and the mean score of each item was 3.21 (SD = 0.85).

**Table 2:** Participants’ scores on each of the scales (*n* = 582).

Scale	Total (mean ± SD)	Item score (mean ± SD)
Career decision-making difficulties	99.34 ± 21.78	3.10 ± 0.68
Lack of readiness	30.67 ± 7.27	3.07 ± 0.73
Lack of information	37.44 ± 9.80	3.12 ± 0.82
Inconsistent information	31.23 ± 8.02	3.12 ± 0.80
Impostor phenomenon	57.58 ± 12.13	3.20 ± 0.67
Fake	25.93 ± 5.99	3.24 ± 0.75
Luck	18.52 ± 4.63	3.09 ± 0.77
Discount	13.13 ± 3.29	3.28 ± 0.82
Psychological resilience	32.11 ± 8.50	3.21 ± 0.85

### 3.4 Correlation analysis

As listed in Table 3, correlation analysis showed that psychological resilience had a significant negative correlation with career decision-making difficulties (*r* = −0.724, *p* < 0.01), and impostor phenomenon had a significant negative correlation with psychological resilience (*r* = −0.608, *p* < 0.01), and had a significant positive correlation with career decision-making difficulties (*r* = 0.700, *p* < 0.01).

**Table 3:** Correlation analysis between variables (*n* = 582).

Variable	1	2	3	4	5	6	7	8	9
1.CDDQ	1								
2. LR	0.806**	1							
3. LI	0.909**	0.592**	1						
4. II	0.874**	0.558**	0.710**	1					
5.IP	0.700**	0.592**	0.601**	0.628**	1				
6. IP (1)	0.621**	0.523**	0.542**	0.551**	0.920**	1			
7. IP (2)	0.603**	0.520**	0.509**	0.543**	0.850**	0.640**	1		
8. IP (3)	0.600**	0.498**	0.515**	0.550**	0.817**	0.670**	0.562**	1	
9. PR	−0.724**	−0.602**	−0.645**	−0.632**	−0.608**	−0.537**	−0.549**	−0.491**	1

CDDQ, career decision-making difficulties; LR, lack of readiness; LI, lack of information; II, inconsistent information; IP, impostor phenomenon; IP(1)-IP(3), fake, luck, discount; PR, psychological resilience. **p < 0.01.

### 3.5 Regression analysis and mediation effect test analysis

In order to examine the mediating effect of psychological resilience in the relationship between impostor phenomenon and career decision-making difficulties, tests of mediation were conducted according to the procedure set by Baron and Kenny (1986). The results of the regression analysis are shown in Table 4. The mediating effect test can be carried out through regression analysis in three steps. In the first step, with socio-demographic variables as controlled variables, a simple regression analysis is carried out with impostor phenomenon as the independent variable and career decision-making difficulties as the dependent variable, to test the coefficient c. In the second step, a simple regression analysis is carried out with impostor phenomenon as the independent variable and psychological resilience as the dependent variable, to test the coefficient a. In the third step, a multiple regression analysis is carried out with impostor phenomenon and psychological resilience as the independent variables and career decision-making difficulties as the dependent variable, to test the coefficient c’ and the coefficient b.

**Table 4:** Regression analysis of the relationship between variables (*n* = 582).

Step	Outcome variable	Predictor variable	*R* ^2^	β	*t*
Step 1	CDDQ	IP	0.488	0.700	23.576***
Step 2	PR	IP	0.369	−0.608	−18.440***
Step 3	CDDQ	IP	0.488	0.411	12.935***
		PR	0.630	−0.474	−14.923***

CDDQ, career decision-making difficulties; IP, impostor phenomenon; PR, psychological resilience. ***p < 0.001.

The results showed that impostor phenomenon is negatively associated with career decision-making difficulties (*β* = 0.700, *p* < 0.001), and negatively associated with psychological resilience (*β* = −0.608, *p* < 0.001). After the introduction of psychological resilience, the influence coefficient (*β* = 0.411, *p* < 0.001) of impostor phenomenon on career decision-making difficulties decreased. This indicated that psychological resilience is a partial mediator in the relationship between impostor phenomenon and career decision-making difficulties.

**Figure 1:**
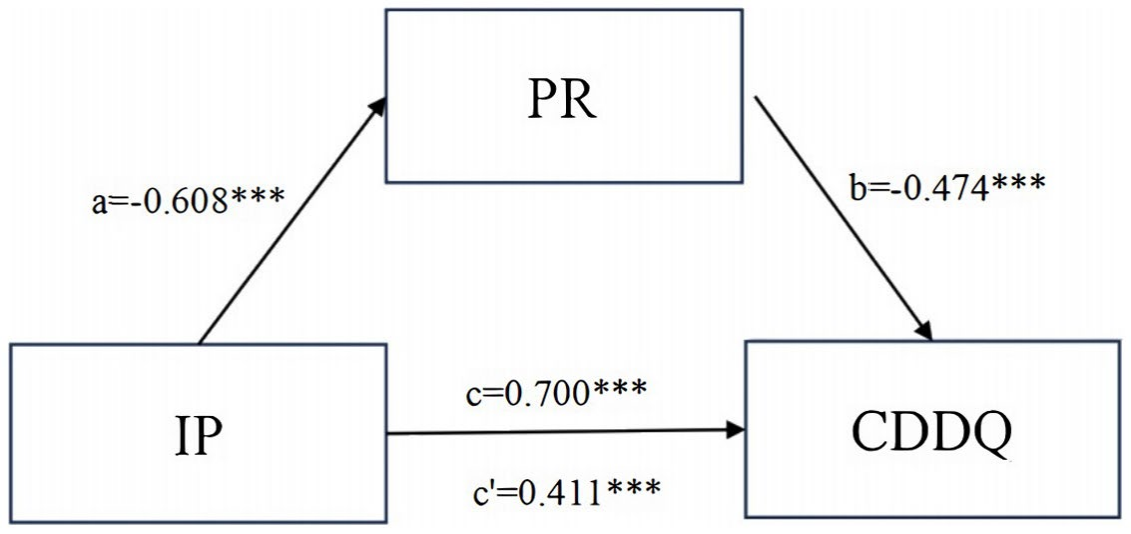
Model of the mediating role of PR between IP and CDDQ (*n* = 582). Career Decision-making Difficulties (CDDQ), Impostor Phenomenon (IP), Psychological Resilience (PR). ***p < 0.001. a, the standardized regression coefficient between IP and PR; b, the standardized regression coefficient between CDDQ and PR; c, the total effect between IP and CDDQ; c’, the direct effect of IP on CDDQ.

Based on the linear regression analysis, both the tendency toward impostor phenomenon and psychological resilience were employed as predictor variables in constructing a structural equation model for career decision-making difficulties among nursing interns. A visualization of the model is displayed in Figure 1.

The results of the mediation effect test analysis are displayed in Table 5. The Bootstrap method was used to test the mediating role of psychological resilience in the mode (Hayes and Scharkow, 2013). After demographic variables were controlled, impostor phenomenon was used as the independent variable, career decision-making difficulties as the dependent variable, and psychological resilience as mediating variables, and the Bootstrap method was used to calculate 95% confidence intervals for each of the 5,000 repeated draws. The value of the direct effect is 0.737, Bootstrap’s 95% CI of total direct effect did not contain 0 [Bootstrap 95% CI: 0.626, 0.850], indicating that the direct effect is established. The value of the indirect effect is 0.518, Bootstrap’s 95% CI of total indirect effect did not contain 0 [Bootstrap 95% CI: 0.430, 0.610], indicating that the indirect effect is established. The mediating effect of psychological resilience accounts for 41.27% of the total effect.

**Table 5:** The mediating effect of psychological resilience between impostor phenomenon and career decision-making difficulties (*n* = 582).

Path	Effect	Boot SE	Boot LLCI	Boot ULCI	Relative effect
Total effect	1.255	0.053	1.151	1.360	100%
Direct effect	0.737	0.057	0.626	0.850	58.73%
Indirect effect	0.518	0.046	0.430	0.610	41.27%

## 4 Discussion

### 4.1 The career decision-making difficulties of nursing interns are at a medium to high level

Our study revealed that the score on the Career Decision Difficulty Scale for nursing interns was 99.34 (SD = 21.78), indicating a moderate to high difficulty in career decision-making. The mean score of 3.10 (SD = 0.68) was higher than the findings for nursing students (Bi et al., 2023). These difficulties can be attributed to various factors, including lack of readiness, information deficits, and inconsistent information, which align with previous research identifying these as common challenges in career decision-making (Kulcsár et al., 2020). It is essential to recognize that career decision-making is a multifaceted process that is influenced by personal, educational, and environmental factors. Moreover, nursing interns often have a limited understanding of the profession’s scope due to their lack of practical experience. This deficiency impedes their grasp of professional roles. They might hold idealized expectations of the nursing field, which often clash with the realities of workplace challenges and pressures, leading to a psychological disconnect. High expectations for medical technology in China often lead to misunderstandings about medical outcomes among patients and their families, as the limitations of medicine are frequently underestimated. These misunderstandings may induce stress and conflict between healthcare providers and patients. Notably, pursuing “medical malpractice” claims for financial compensation heightens these tensions and erodes nursing interns’ sense of security and trust within their professional environment.

In addition, this study found that two of the three dimensions of career decision-making difficulties, namely “inconsistent information” and “lack of information” had mean scores higher than the overall scale mean. The dimension of “inconsistent information” encompasses “unreliable information,” “internal conflicts,” and “external conflicts” (Gati et al., 2000), indicating that nursing interns may experience conflicts between their personal beliefs and career expectations, as well as disagreements with parents during their career exploration. These conflicts could significantly influence their career choices and personal development. On the other hand, the “lack of information” dimension includes issues such as an inadequate understanding of the decision-making process, limited self-awareness, insufficient information about career options, and limited access to relevant resources. This also explains the higher career decision-making difficulties scores of female nursing interns from rural areas. Lacking information and guidance, they may experience uncertainty and anxiety about their career decisions. The nursing interns typically seek guidance in career planning, clarification on decision-making essentials, and assistance in aligning their circumstances with career requirements (Wei et al., 2021). Additionally, the short internship period and academic responsibilities limit access to career information, leading to a need for more awareness about available options (Schnoes et al., 2018). It is suggested that educators and hospital administrators continuously evaluate the career guidance needs of nursing interns. They should also arrange lectures on employment issues, enhance understanding of the challenges and offer targeted guidance. These efforts are aimed at enhancing their understanding of career decision-making, ultimately clarifying their future career paths (Zeng et al., 2022).

### 4.2 The impostor phenomenon of nursing interns is at a moderate level

The results of this study indicate a moderate level of impostor phenomenon among nursing interns, with a mean score of 57.58 (SD = 12.13), slightly above the scale’s midpoint of 54. Similar trends have been observed in North America (Chakraverty, 2024) and India (Sawant et al., 2023). This phenomenon can be attributed to various factors. Firstly, limited exposure to real-life clinical scenarios can erode their confidence, as they may need more opportunities to practice and refine their skills in authentic healthcare settings. This lack of practical experience can leave them unprepared and doubtful of their abilities. Secondly, the demanding nature of healthcare environments and the emotional toll of nursing can exacerbate work-related stress. In Chinese hospitals, rigorous and frequent nursing quality supervision can make nursing interns feel unable to cope. They may perceive their performance as falling short of these high standards. Moreover, strained nurse–patient relationships can hinder effective communication, problem-solving, and critical nursing skills. The inability to communicate can decrease confidence and contribute to the impostor phenomenon. Another significant factor is the lack of support and mentorship during clinical practice (Bahari et al., 2022). Nursing interns may feel more secure and confident in their capabilities with proper guidance. Negative feedback in such an environment has the potential to intensify self-doubt and reinforce the impostor phenomenon. To address this, educators and hospital administrators should prioritize the psychological well-being of nursing interns. It can be achieved through regular symposiums, enhanced psychological support, and promotion of positive psychological traits. Nursing interns should also be encouraged to view negative evaluations constructively and cultivate a growth mindset, which can mitigate the fear of negative feedback and diminish the impostor phenomenon (Xia et al., 2020).

### 4.3 Discussion of the four research hypotheses

This study investigates the role of psychological resilience in mediating the relationship between nursing interns’ impostor phenomenon and career decision-making difficulties. The research design is based on four hypotheses, each of which discussed in detail.

Within the scope of the first hypothesis of this research, the negative correlation between nursing interns’ impostor phenomenon and psychological resilience was examined. Existing research suggests that the impostor phenomenon generates associations related to psychological well-being through related behaviors and perceptions (Brauer and Wolf, 2016). When nursing interns experience the impostor phenomenon, they may doubt their abilities and accomplishments, leading to decreased self-confidence and an inability to internalize their successes. This can result in increased anxiety, depression, and psychological distress, which in turn can undermine their resilience. Medical students who frequently experience this phenomenon are more likely to suffer from severe depression and entertain suicidal thoughts than their counterparts (Levant et al., 2020). Additionally, a growth mindset can alleviate the negative impacts on the academic performance of disadvantaged groups (Claro et al., 2016). The impostor phenomenon can be seen as an extension of this dynamic, where individuals with a fixed mindset may be more susceptible to internalizing negative stereotypes and perceiving themselves as impostors due to their belief in the immutability of their abilities. This mindset can impede their academic success by reducing their willingness to seek help, or take on challenging tasks. Conversely, a growth mindset being positively related to resilience and negatively related to the impostor phenomenon (Ibrahim et al., 2022).

Within the scope of the second hypothesis of this research, the negative correlation between nursing interns’ psychological resilience and career decision-making difficulties was examined. Specifically, individuals with higher psychological resilience tend to have greater self-confidence and self-efficacy, expanding their career options career options (Zhou W. et al., 2024). Self-confidence is considered a critical element in the career decision-making process. This trait empowers individuals to be more resolute and decisive, reducing the challenges of indecisiveness (Santos et al., 2018). Moreover, individuals possessing high psychological resilience are adept at maintaining composure under stress, navigating career decision-making challenges effectively, and identifying dependable information sources, all essential for managing various obstacles during the career decision-making process (Wu et al., 2020).

Within the scope of the third hypothesis of this research, the positive correlation between nursing interns’ impostor phenomenon and career decision-making difficulties was examined. Nursing interns, who are at a critical juncture in their professional development, often grapple with self-doubt and feelings of inadequacy despite their academic and clinical achievements. These emotions can significantly impact their career decision-making processes, leading to difficulties in choosing a specialization, pursuing further education, or deciding on an employment setting. Specifically, a lower incidence of the impostor phenomenon correlates with reduced challenges in making career decisions. Continuous self-doubt and denial of success may lead individuals to experience helplessness and anxiety in the workplace or academic settings (Ibrahim et al., 2022). The impostor phenomenon undermines nursing interns’ self-perceptions, leading to an underestimation of their abilities (Zanchetta et al., 2020). This underestimation can make them more vulnerable to external pressures during career decision-making, leading to negative behaviors like procrastination and avoidance (Anghel and Gati, 2021). Additionally, cognitive biases impede their capacity to collect and analyze information, ultimately hindering their ability to make objective and comprehensive career decisions (Soprano et al., 2024).

Within the scope of the fourth hypothesis of this research, the mediating role of psychological resilience in the relationship between nursing interns’ impostor phenomenon and career decision-making difficulties was examined. Psychological resilience explained more than 40% of the variation in the total effect, demonstrating its important role in this process. This percentage points out that psychological resilience appears to mediate part of the effect of the impostor phenomenon on career decision-making difficulties, suggesting that variations in resilience may account for some of the observed relationship between these two variables. By adapting to the challenges and uncertainties associated with career progression, individuals with higher psychological resilience may experience less decision-making difficulty and more successful career planning. They effectively acknowledge reality, accurately identify challenges, and devise solutions when confronted with unfamiliar and complex tasks, rather than resorting to denial or avoidance (Shin and Kelly, 2015). In contrast, individuals with low psychological resilience frequently face psychological challenges in unfamiliar clinical environments and complex interpersonal situations, resulting in negative emotions such as anxiety and worry (Wenxia et al., 2022). If these emotions are not effectively managed, they can gradually erode self-efficacy, and ultimately influence job satisfaction, burnout and turnover intention (Labrague and De los Santos, 2020). Psychological resilience has been shown to play a crucial role in determining psychological health during critical periods, such as the transition from student to professional nurse. It serves as a protective factor against stress and burnout, which are common among nursing students due to the intense and emotionally charged nature of their work. Psychological resilience is a dynamic and influential process that can potentially mitigate the impact of the impostor phenomenon on the career decisions of nursing interns. Its relevance is further underscored by its role in coping with the emotional labor inherent in nursing and its potential to be developed and enhanced through targeted interventions.

## 5 Conclusion and suggestions

This study examined the mediating role of psychological resilience between the impostor phenomenon and career decision-making difficulties among nursing interns. Four hypotheses were proposed and tested. The analyses supported the acceptance of all four hypotheses, presenting significant challenges and research questions for scholars in related fields. These findings provide valuable insights for nursing educators, hospital administrators, and nursing interns.

Nursing educators should deepen students’ knowledge of the nursing field through additional vocational education courses, lectures, and symposia. These efforts aim to ignite a passion for nursing and boost interns’ self-assurance. Furthermore, incorporating psychological preparation courses into internship curricula can equip students with essential skills for emotional management, stress coping, and effective communication. Such courses are critical in fostering positive psychological anticipation, nurturing a growth mindset, and stimulating proactive engagement in career exploration.

Hospital administrators must prioritize optimizing the nursing internship environment by providing comprehensive guidance and support. Additionally, it is essential to provide qualified mentors who can assist them with a sense of responsibility and professional attitude. These strategies aim to alleviate work-related stress and empower interns to gain a deeper insight into their strengths and interests.

For nursing interns, cultivating a confident and optimistic psychological disposition is essential. Exploring internal and external environments can significantly strengthen motivation toward career objectives. They view challenges as surmountable during career decision-making processes. Such a proactive approach facilitates the transition from nursing student to professional nurse.

We emphasize that while we can observe associations and propose potential mediation, we cannot infer causality or the direction of effects based on our data. Future research should focus on the long-term effects of the impostor phenomenon and psychological resilience on career development in nursing. Investigating the efficacy of interventions aimed at enhancing psychological resilience and mitigating the impostor phenomenon could provide valuable strategies for supporting nursing interns. Additionally, cross-cultural studies could offer insights into the generalizability of our findings and the effectiveness of different approaches across diverse populations.

## 6 Limitations

This study has the following limitations. First of all, the cross-sectional design limits our ability to establish causal relationships between the variables, future research should employ longitudinal designs to further explore these relationships and determine potential causal pathways. While our study suggests that psychological resilience (PR) mediates the relationship between impostor phenomenon (IP) and career decision-making difficulties (CDDQ), it is possible that PR could be the predictor variable, with IP mediating the relationship between PR and CDDQ. This alternative causal pathway cannot be ruled out due to the cross-sectional nature of our data. Secondly, the use of convenience sampling may have introduced selection bias, as our sample may not be representative of all nursing interns. This limitation affects the generalizability of our findings and suggests that our results may not be applicable to nursing interns in different contexts or settings. Thirdly, the reliance on self-reported data can introduce biases due to the participants’ possible inaccuracies or selective memory. Future research should target a more extensive and diverse sample from various regions and adopt a longitudinal design to understand causal relationships better and enhance result reliability.

## Data Availability

The original contributions presented in the study are included in the article/supplementary material, further inquiries can be directed to the corresponding author.
