# Supplementary material for: The relationship between impostor phenomenon and career decision-making difficulties among nursing interns: the mediating role of psychological resilience
Source: Front Psychol. 2024 Nov 27;15:1484708. doi: 10.3389/fpsyg.2024.1484708 (PMC11632309; doi:10.3389/fpsyg.2024.1484708)
Supplement: Supplementary file 1 [file Data_Sheet_1.PDF]

Informed Consent

Dear nursing interns:

Hello! In order to understand the current situation of nursing interns' career decision-making difficulties and impostor phenomenon, and to promote the benign development of intern nursing students' career, we would like to invite you to participate in this research, and we kindly ask you to fill in the information in accordance with the guidelines patiently and truthfully, as the information you fill in is crucial to the research. Thank you very much for your support!

This questionnaire study involves the Career Decision Difficulty Scale, the Clance Impostor Phenomenon Scale, and Psychological Resilience Scale for intern nursing students. The questionnaire will take approximately 5 minutes. Your participation in this survey is completely voluntary and at no cost to you, and you may decide at any time not to participate in this survey. We hereby solemnly promise you that this questionnaire is anonymous and there is no right or wrong answer. All information collected is for statistical analysis only, we will keep it strictly confidential to ensure your privacy, please feel free to answer.

If you have any questions about this study, please contact us. Email: 2717595033@qq.com

Thank you for your cooperation and strong support for this study!

I have read this informed consent form and volunteer to participate in this study.

☐Agree

☐Disagree

1.How old are you?

○19 ○20 ○21

○22 ○23 ○24

2. What is your gender？

○Male ○Female

3. What is your registered residence?

○Urban ○Rural

4. What is your duration of internship？

○Less than 8 months ○8-9 months ○More than 9 months

5. Do you have a clear intention to work?

○Yes ○No

Guideline: For each of the following statements, please select the one that most closely matches you based on the most recent week. 1~5 stands for Not at all, Somewhat not, Not sure, Somewhat, Very much.

6. I know I have to choose a career, but now I have no desire to make a decision.

Strongly disagree ○1 ○2 ○3 ○4 ○5 Strongly agree

7. Work is not the most important thing in life, so issues like choosing a career don't worry me too much.

Strongly disagree ○1 ○2 ○3 ○4 ○5 Strongly agree

8. I don't think it's necessary to choose a career right now because I will naturally make the right career choice over time.

Strongly disagree ○1 ○2 ○3 ○4 ○5 Strongly agree

9. Making decisions is usually difficult for me.

Strongly disagree ○1 ○2 ○3 ○4 ○5 Strongly agree

10. I usually think I need the approval and support of a professional or someone I trust for

my decisions.

Strongly disagree ○1 ○2 ○3 ○4 ○5 Strongly agree

11. I usually worry about failing.

Strongly disagree ○1 ○2 ○3 ○4 ○5 Strongly agree

12. I like to do things my own way.

Strongly disagree ○1 ○2 ○3 ○4 ○5 Strongly agree

13. I expect that entering the career I select will also solve my personal problems.

Strongly disagree ○1 ○2 ○3 ○4 ○5 Strongly agree

14. I believe there is only one career that suits me.

Strongly disagree ○1 ○2 ○3 ○4 ○5 Strongly agree

15. I expect that through the career I select I will satisfy all my wishes.

Strongly disagree ○1 ○2 ○3 ○4 ○5 Strongly agree

16. I believe that a career selection is a one-time choice and a life-long commitment.

Strongly disagree ○1 ○2 ○3 ○4 ○5 Strongly agree

17. I always do what I'm told, even if it's not to my own liking.

Strongly disagree ○1 ○2 ○3 ○4 ○5 Strongly agree

18. I find it difficult to make a decision about the career because: I do not know what steps I have to take.

Strongly disagree ○1 ○2 ○3 ○4 ○5 Strongly agree

19. I find it difficult to make a decision about the career because:I do not know what factors to take into consideration.

Strongly disagree ○1 ○2 ○3 ○4 ○5 Strongly agree

20. I find it difficult to make a decision about the career because: I don't know how to combine the information I have about myself with the information I have about different careers.

Strongly disagree ○1 ○2 ○3 ○4 ○5 Strongly agree

21. I find it difficult to make a decision about the career because: I still do not know which occupations are interesting to me.

Strongly disagree ○1 ○2 ○3 ○4 ○5 Strongly agree

22. I find it difficult to make a decision about the career because:I am not sure about my career preferences yet (for example, what kind of a relationship I want with people, which working environment I prefer?).

Strongly disagree ○1 ○2 ○3 ○4 ○5 Strongly agree

23. I find it difficult to make a decision about the career because:I do not have enough information about my competencies (for example, numerical ability, verbal skills) and/or about my personality traits (for example, persistence, initiative, patience).

Strongly disagree ○1 ○2 ○3 ○4 ○5 Strongly agree

24. I find it difficult to make a decision about the career because:I do not know what my abilities and/or personality traits will be like in the future.

Strongly disagree ○1 ○2 ○3 ○4 ○5 Strongly agree

25. I find it difficult to make a decision about the career because:I don't know much about the kinds of occupations and training programs available.

Strongly disagree ○1 ○2 ○3 ○4 ○5 Strongly agree

26. I find it difficult to make a decision about the career because:I do not have enough information about the characteristics of the occupations and/or training programs that interest me.

Strongly disagree ○1 ○2 ○3 ○4 ○5 Strongly agree

27. I find it difficult to make a decision about the career because:I don't know what careers will look like in the future.

Strongly disagree ○1 ○2 ○3 ○4 ○5 Strongly agree

28. I find it difficult to make a decision about the career because: I do not know how to obtain additional information about myself (for example, about my abilities or my personality traits).

Strongly disagree ○1 ○2 ○3 ○4 ○5 Strongly agree

29. I find it difficult to make a decision about the career because:I do not know how to obtain accurate and updated information about the existing occupations or about their characteristics.

Strongly disagree ○1 ○2 ○3 ○4 ○5 Strongly agree

30. I find it difficult to make a decision about the career because:I don't know which occupations I'm interested in.

Strongly disagree ○1 ○2 ○3 ○4 ○5 Strongly agree

31. I find it difficult to make a decision about the career because:I constantly change my career preference (for example, I want to be self-employed and sometimes I want to be an employee).

Strongly disagree ○1 ○2 ○3 ○4 ○5 Strongly agree

32. I find it difficult to make a decision about the career because:I have contradictory data about the existence or the characteristics of a particular occupation.

Strongly disagree ○1 ○2 ○3 ○4 ○5 Strongly agree

33. I find it difficult to make a decision about the career because:I have been given conflicting information about certain careers, training programs, and employers.

Strongly disagree ○1 ○2 ○3 ○4 ○5 Strongly agree

34. I find it difficult to make a decision about the career because:I'm equally attracted by a number of careers and it is difficult for me to choose among them.

Strongly disagree ○1 ○2 ○3 ○4 ○5 Strongly agree

35. I find it difficult to make a decision about the career because:the career, training pro-

gram or employer that would accept me is not to my liking (the other person sees me, but I don't see the other person).

Strongly disagree ○1 ○2 ○3 ○4 ○5 Strongly agree

36. I find it difficult to make a decision about the career because:the occupation I am interested in involves a certain characteristic that bother me (for example, I am interested in medicine, but I do not want to study for so many years).

Strongly disagree ○1 ○2 ○3 ○4 ○5 Strongly agree

37. I find it difficult to make a decision about the career because:my career preferences (wants) can't be included in the same career and I don't want to give them up (e.g. I want to be a freelancer but want a steady income).

Strongly disagree ○1 ○2 ○3 ○4 ○5 Strongly agree

38. I find it difficult to make a decision about the career because:my skills and abilities do not match those required by the occupation I am interested in.

Strongly disagree ○1 ○2 ○3 ○4 ○5 Strongly agree

39. I find it difficult to make a decision about the career because:People who are important to me do not agree with the career options I am considering and/or the career characteristics I desire.

Strongly disagree ○1 ○2 ○3 ○4 ○5 Strongly agree

40. I find it difficult to make a decision about the career because:There are contradictions between the recommendations made by different people who are important to me about the career that suits me or about what career characteristics should guide my decisions.

Strongly disagree ○1 ○2 ○3 ○4 ○5 Strongly agree

41. I avoid evaluations if possible and have a dread of others evaluating me.

Not at all true ○1 ○2 ○3 ○4 ○5 Very true

42.When people praise me for something I’ve accomplished, I’m afraid I won’t be able

to live up to their expectations of me in the future.

Not at all true ○1 ○2 ○3 ○4 ○5 Very true

43. I sometimes think I obtained my present position or gained my present success because I happened to be in the right place at the right time or knew the right people.

Not at all true ○1 ○2 ○3 ○4 ○5 Very true

44. I’m afraid people important to me may find out that I’m not as capable as they think I am.

Not at all true ○1 ○2 ○3 ○4 ○5 Very true

45. I tend to remember the incidents in which I have not done my best more than those times I have done my best.

Not at all true ○1 ○2 ○3 ○4 ○5 Very true

46. I rarely do a project or task as well as I’d like to do it.

Not at all true ○1 ○2 ○3 ○4 ○5 Very true

47. Sometimes I feel or believe that my success in my life or in my job has been the result of some kind of error.

Not at all true ○1 ○2 ○3 ○4 ○5 Very true

48. It’s hard for me to accept compliments or praise about my intelligence or accomplishments.

Not at all true ○1 ○2 ○3 ○4 ○5 Very true

49. At times, I feel my success has been due to some kind of luck.

Not at all true ○1 ○2 ○3 ○4 ○5 Very true

50. I’m disappointed at times in my present accomplishments and think I should have accomplished much more.

Not at all true ○1 ○2 ○3 ○4 ○5 Very true

51. Sometimes I’m afraid others will discover how much knowledge or ability I really lack

Not at all true ○1 ○2 ○3 ○4 ○5 Very true

52. I'm often afraid that I may fail at a new assignment or undertaking even though I generally do well at what I attempt.

Not at all true ○1 ○2 ○3 ○4 ○5 Very true

53. When I've succeeded at something and received recognition for my accomplishments, I have doubts that I can keep repeating that success.

Not at all true ○1 ○2 ○3 ○4 ○5 Very true

54. If I receive a great deal of praise and recognition for something I've accomplished, I tend to discount the importance of what I've done.

Not at all true ○1 ○2 ○3 ○4 ○5 Very true

55. I often compare my ability to those around me and think they may be more intelligent than I am.

Not at all true ○1 ○2 ○3 ○4 ○5 Very true

56. I often worry about not succeeding with a project or examination, even though others around me have considerable confidence that I will do well.

Not at all true ○1 ○2 ○3 ○4 ○5 Very true

57. If I'm going to receive a promotion or gain recognition of some kind, I hesitate to tell others until it is an accomplished fact.

Not at all true ○1 ○2 ○3 ○4 ○5 Very true

58. I feel bad and discouraged if I'm not "the best" or at least "very special" in situations that involve achievement.

Not at all true ○1 ○2 ○3 ○4 ○5 Very true

Guideline: For each of the following statements, please select the one that best describes you based on how you have been doing in the past month. 0 to 4 represents Never, Rarely, Sometimes, Often, and Always.

59. I am able to adapt to any change required by the situation.

Never ○0 ○1 ○2 ○3 ○4 Always

60. At work, I can deal with whatever comes.

Never ○0 ○1 ○2 ○3 ○4 Always

61. Even when facing work hardships, I try to see the humorous side of problems.

Never ○0 ○1 ○2 ○3 ○4 Always

62. Coping with work hardships can strengthen me.

Never ○0 ○1 ○2 ○3 ○4 Always

63. I tend to quickly bounce back after work hardships.

Never ○0 ○1 ○2 ○3 ○4 Always

64. I can achieve work goals despite obstacles.

Never ○0 ○1 ○2 ○3 ○4 Always

65. I am not easily discouraged by work failure.

Never ○0 ○1 ○2 ○3 ○4 Always

66. At work, I think of myself as a strong person.

Never ○0 ○1 ○2 ○3 ○4 Always

67. I can handle unpleasant feelings about work.

Never ○0 ○1 ○2 ○3 ○4 Always

68. I can stay focused even when under pressure.

Never ○0 ○1 ○2 ○3 ○4 Always
