# Supplementary material for: The relationship between impostor phenomenon and career decision-making difficulties among nursing interns: the mediating role of psychological resilience
Source: Front Psychol. 2024 Nov 27;15:1484708. doi: 10.3389/fpsyg.2024.1484708 (PMC11632309; doi:10.3389/fpsyg.2024.1484708)
Supplement: Supplementary file 2 [file Data_Sheet_2.PDF]

知情同意书

亲爱的同学们：

您们好！为了解当前护理实习生职业决策困难与自我能力否定倾向现状，促进实习护生职业生涯良性发展，特邀您参加本次调研，恳请您按照指导语耐心如实填写，您所填资料对研究至关重要。万分感谢您的支持！

本问卷研究涉及实习护生职业决策困难量表、自我能力否定倾向量表及心理弹性量表等内容。问卷调查大约在 3 分钟左右。您是否参加本次调查完全是自愿的，也不需要您承担任何费用，您可以在任何时候决定不参与本次调查。在此郑重向您承诺，本问卷不记名，答案均无对错之分。所有收集到的信息仅供统计分析之用，我们将对其严格保密，确保您的隐私，敬请放心回答。

如对本次研究有任何疑问，请联系我们。电子邮箱：2717595033@qq.com

感谢您对本次研究的配合与大力支持！

我已阅读这份知情同意书，并自愿参加这项研究。

□同意

□不同意

1. 您的年龄：

〇19　〇20　〇21

〇22　〇23　〇24

2. 您的性别：

〇男　〇女

3. 生源地

〇城镇　〇农村

4. 已实习时长

〇未满 8 个月　〇已满 8-9 个月　〇9 个月以上

5. 您目前是否有明确的工作意向？

〇是　〇否

指导语：请您根据最近一周的情况，对下面每个阐述，选出最符合您的一项。1~5 分别代表完全不符合、有些不符合、不清楚、有些符合、非常符合。

6. 我知道我必须选择一个职业，但现在我还没有做决定的意愿。

完全不符合 〇1　〇2　〇3　〇4　〇5　非常符合

7. 工作并不是人生中最重要的事情，所以选择职业这种问题并不太让我担心。

完全不符合 〇1　〇2　〇3　〇4　〇5　非常符合

8. 我认为不必现在就选择一个职业，因为随着时间的推移，我自然会做出正确的职业选择。

完全不符合 〇1　〇2　〇3　〇4　〇5　非常符合

9. 对我而言，做决定通常是困难的。

完全不符合 〇1　〇2　〇3　〇4　〇5　非常符合

10. 我通常觉得自己的决定需要专业人士或自己信赖的人的认可和支持。

完全不符合 〇1　〇2　〇3　〇4　〇5　非常符合

11. 我通常害怕失败。

完全不符合 〇1 〇2 〇3 〇4 〇5 非常符合

12. 我喜欢以我自己的方式行事。

完全不符合 〇1 〇2 〇3 〇4 〇5 非常符合

13. 我希望通过从事我所选择的职业,能够解决我的其他个人问题(如人际关系、家庭、感情等)。

完全不符合 〇1 〇2 〇3 〇4 〇5 非常符合

14. 我认为只有一个职业适合我。

完全不符合 〇1 〇2 〇3 〇4 〇5 非常符合

15. 我希望通过从事我所选择的职业，能够实现所有的人生愿望。

完全不符合 〇1 〇2 〇3 〇4 〇5 非常符合

16. 我认为职业选择是一次性的决定和终生的承诺(一旦选择一份职业就不能再考虑其他选择，也不能更换)。

完全不符合 〇1 〇2 〇3 〇4 〇5 非常符合

17. .我总是按照别人的吩咐去做事，即使这样做并不符合我自己的意愿。

完全不符合 〇1 〇2 〇3 〇4 〇5 非常符合

18. 我觉得职业选择是一件困难的事，因为我不知道应该采取什么样的步骤。

完全不符合 〇1 〇2 〇3 〇4 〇5 非常符合

19. 我觉得职业选择是一件困难的事，因为我不知道应该考虑哪些因素。

完全不符合 〇1 〇2 〇3 〇4 〇5 非常符合

20. 我觉得职业选择是一件困难的事,因为我不知道如何将自身的情况(如自己适合做什么)和各种不同职业的信息(如不同职业对人的要求)结合起来考虑。

完全不符合 〇1 〇2 〇3 〇4 〇5 非常符合

21. 我觉得职业选择是一件困难的事，因为我还不知道哪些职业是我所感兴趣的。

完全不符合 〇1 〇2 〇3 〇4 〇5 非常符合

22. 我觉得职业选择是一件困难的事，因为我还不能确定自己的职业偏好(如，我希望

与他人建立什么样的人际关系，我喜好怎样的工作环境等)。

完全不符合 〇1 〇2 〇3 〇4 〇5 非常符合

23. 我觉得职业选择是一件困难的事，因为我还不太了解自己的能力(如数学能力、语言表达能力)和性格特征(如毅力，主动性，耐心等)。

完全不符合 〇1 〇2 〇3 〇4 〇5 非常符合

24. 我觉得职业选择是一件困难的事，因为我不知道将来我的能力和性格特征会是什么样的。

完全不符合 〇1 〇2 〇3 〇4 〇5 非常符合

25. 我觉得职业选择是一件困难的事，因为我对现有职业和培训项目的种类不太了解。

完全不符合 〇1 〇2 〇3 〇4 〇5 非常符合

26. 我觉得职业选择是一件困难的事，因为我对自己感兴趣的职业和培训项目的特点不太了解(如市场需求、一般收入、晋升的可能性、培训项目补贴等)

完全不符合 〇1 〇2 〇3 〇4 〇5 非常符合

27. 我觉得职业选择是一件困难的事，因为我不知道将来的职业会是什么样的，如职业的发展前景、将来的市场需求如何。

完全不符合 〇1 〇2 〇3 〇4 〇5 非常符合

28. 我觉得职业选择是一件困难的事，因为我不知道怎样能更加清楚地了解自己(如怎样了解我的能力和性格特征)。

完全不符合 〇1 〇2 〇3 〇4 〇5 非常符合

29. 我觉得职业选择是一件困难的事，因为我不知道如何获得关于现有职业、培训项目、用人单位的准确和最新信息。

完全不符合 〇1 〇2 〇3 〇4 〇5 非常符合

30. 我觉得职业选择是一件困难的事，因为我不知道我对哪些职业感兴趣。

完全不符合 〇1 〇2 〇3 〇4 〇5 非常符合

31. 我觉得职业选择是一件困难的事，因为我经常变换自己的职业偏好(如有时我想自

己当老板，而有时我只想受雇于人)。

完全不符合 〇1 〇2 〇3 〇4 〇5 非常符合

32. 我觉得职业选择是一件困难的事，因为我获得的关于自己能力和性格特征的信息有互相矛盾的地方(如我认为自己是个有耐心的人，而其他人并不这么认为)。

完全不符合 〇1 〇2 〇3 〇4 〇5 非常符合

33. 我觉得职业选择是一件困难的事，因为我所获得的某种职业、培训项目、用人单位的信息有互相矛盾的地方。

完全不符合 〇1 〇2 〇3 〇4 〇5 非常符合

34. 我觉得职业选择是一件困难的事，因为若干个职业同样吸引着我，从它们之中选择一个有些困难。

完全不符合 〇1 〇2 〇3 〇4 〇5 非常符合

35. 我觉得职业选择是一件困难的事，因为能接受我的职业、培训项目或用人单位却不是我所喜欢的(对方看中了我，但我看不中对方)。

完全不符合 〇1 〇2 〇3 〇4 〇5 非常符合

36. 我觉得职业选择是一件困难的事，因为我感兴趣的职业包含一些我不喜欢的职业性质(如我对某职业感兴趣，但那需要学习很多年，而我不愿意学习那么多年)。

完全不符合 〇1 〇2 〇3 〇4 〇5 非常符合

37. 我觉得职业选择是一件困难的事，因为我的职业偏好(想要的东西)不能被包含在同一个职业里，而我又不想放弃它们(如我想成为一个自由职业者，但又希望有一份稳定的收入)。

完全不符合 〇1 〇2 〇3 〇4 〇5 非常符合

38. 我觉得职业选择是一件困难的事，因为我不具备我所感兴趣的职业所要求的能力。

完全不符合 〇1 〇2 〇3 〇4 〇5 非常符合

39. 我觉得职业选择是一件困难的事，因为对我而言比较重要的人(如父母、朋友)并不认同我正在考虑的职业或我所想要的职业特质。

完全不符合 〇1 〇2 〇3 〇4 〇5 非常符合

40. 我觉得职业选择是一件困难的事，因为对我而言比较重要的人对什么职业适合我或选择职业时应该考虑哪些方面这些问题上存在着不同看法。

完全不符合 〇1 〇2 〇3 〇4 〇5 非常符合

41. 我尽量避免评价别人，并且害怕别人评价我。

完全不符合 〇1 〇2 〇3 〇4 〇5 非常符合

42. 当人们因为我完成了某件事而称赞我的时候，我担心我将来会辜负他们对我的期望。

完全不符合 〇1 〇2 〇3 〇4 〇5 非常符合

43. 我有时会想，我之所以能得到现在的职位或取得现在的成就，是因为我碰巧在对的时间出现在对的地方，或者认识了对的人。

完全不符合 〇1 〇2 〇3 〇4 〇5 非常符合

44. 我害怕那些对我重要的人会发现我没有他们想象的那么能干。

完全不符合 〇1 〇2 〇3 〇4 〇5 非常符合

45. 比起那些我竭尽全力做的事情，我更容易记住那些我没有尽最大努力去做的事情。

完全不符合 〇1 〇2 〇3 〇4 〇5 非常符合

46. 我很少把一个项目或任务做得像我想做的那样好。

完全不符合 〇1 〇2 〇3 〇4 〇5 非常符合

47. 有时我认为我在生活或工作上的成功是由于某种差错。

完全不符合 〇1 〇2 〇3 〇4 〇5 非常符合

48. 我很难真正认同别人对我聪明才智或成就的赞扬。

完全不符合 〇1 〇2 〇3 〇4 〇5 非常符合

49. 有时，我觉得我的成功是由于某种运气。

完全不符合 〇1 〇2 〇3 〇4 〇5 非常符合

50. 有时我对自己目前的成就感到失望，认为自己本应取得更大的成就。

完全不符合 〇1 〇2 〇3 〇4 〇5 非常符合

51. 有时候我害怕别人会发现我真正缺乏多少知识或能力。

完全不符合 〇1 〇2 〇3 〇4 〇5 非常符合

52. 我经常担心我不能胜任新的任务或工作，即使我通常都做的很好。

完全不符合 〇1 〇2 〇3 〇4 〇5 非常符合

53. 当我在某件事上取得了成功，并获得了别人对我成就的认可，我会担心自己是否能继续取得成功。

完全不符合 〇1 〇2 〇3 〇4 〇5 非常符合

54. 如果我在某件事情上获得了极大的赞扬和认可，我往往会低估自己所做事情的重要性。

完全不符合 〇1 〇2 〇3 〇4 〇5 非常符合

55. 我经常拿自己的能力和周围的人比较，觉得他们可能比我更聪明。

完全不符合 〇1 〇2 〇3 〇4 〇5 非常符合

56. 我经常担心我不能在某个任务或考试中取得成功，尽管我周围的人都相信我能做好。

完全不符合 〇1 〇2 〇3 〇4 〇5 非常符合

57. 如果我将获得晋升或某种认可，直到这成为一个既成事实，我才愿意告诉别人。

完全不符合 〇1 〇2 〇3 〇4 〇5 非常符合

58. 当我在某方面取得成就时，如果我不是“最好的”或者“非常特别的”，我会感到糟糕和沮丧。

完全不符合 〇1 〇2 〇3 〇4 〇5 非常符合

指导语：请根据过去一个月您的情况，对下面每个阐述，选出最符合您的一项。0~4 分别代表从不这样、很少这样、有时这样、经常这样、总是这样。

59. 当发生变化时，我能灵活应对。

从不这样 〇0 〇1 〇2 〇3 〇4 总是这样

60. 遇到困难时，我可以处理。

从不这样 ○0 ○1 ○2 ○3 ○4 总是这样

61. 面对问题时，我能幽默应对。

从不这样 ○0 ○1 ○2 ○3 ○4 总是这样

62. 透过经验的积累，使我变得更加坚强。

从不这样 ○0 ○1 ○2 ○3 ○4 总是这样

63. 经历生病或苦难后，我的复原能力很强。

从不这样 ○0 ○1 ○2 ○3 ○4 总是这样

64. 即使遇到障碍，我也可以实现目标。

从不这样 ○0 ○1 ○2 ○3 ○4 总是这样

65. 我不会因失败而沮丧。

从不这样 ○0 ○1 ○2 ○3 ○4 总是这样

66. 面临生活中挑战时，我认为我是个坚强的人。

从不这样 ○0 ○1 ○2 ○3 ○4 总是这样

67. 我有能力处理不愉快的感觉，例如生气。

从不这样 ○0 ○1 ○2 ○3 ○4 总是这样

68. 面临压力时，我能聚精会神思考问题。

从不这样 ○0 ○1 ○2 ○3 ○4 总是这样
